# Depressive Symptoms Are Associated with More Hopelessness among White than Black Older Adults

**DOI:** 10.3389/fpubh.2016.00082

**Published:** 2016-05-04

**Authors:** Shervin Assari, Maryam Moghani Lankarani

**Affiliations:** ^1^Department of Psychiatry, University of Michigan, Ann Arbor, MI, USA; ^2^Center for Research on Ethnicity, Culture and Health (CRECH), School of Public Health, University of Michigan, Ann Arbor, MI, USA; ^3^Medicine and Health Promotion Institute, Tehran, Iran

**Keywords:** ethic groups, race, ethnicity, depression, depressive symptoms, hopelessness

## Abstract

**Background:**

Hopelessness is a core component of depression. Our information is, however, very limited on ethnic variations in the magnitude of the link between depression and hopelessness. Using a national sample of older adults in United States, we compared Blacks and Whites for the magnitude of the association between depressive symptoms and hopelessness.

**Methods:**

With a cross-sectional design, we used baseline data of the Religion, Aging, and Health Survey, 2001. Linear regression models were used for data analysis. Depressive symptoms (CES-D) and hopelessness were conceptualized as independent and dependent variables in different models, respectively. Demographic factors (age and gender), socioeconomic status (education and marital status), and health (self-rated health) were covariates. Ethnicity was the moderator.

**Results:**

In the pooled sample, higher depressive symptoms were predictive of hopelessness, above and beyond all covariates. We also found significant interactions suggesting that the association between depressive symptoms and hopelessness is weaker among Blacks compared to Whites. In ethnic-specific models, there were significant associations between depressive symptoms and hopelessness among Whites but not Blacks.

**Conclusion:**

Depressive symptoms accompany more hopelessness among Whites than Blacks. This finding may explain why Blacks with depression have a lower tendency to commit suicide. Future research should test whether or not Whites with depression better respond to psychotherapies and cognitive behavioral therapies that focus on hope enhancement. This finding may explain differential correlates of depression based on race and ethnicity.

## Introduction

Hopelessness is associated with a wide range of negative mood states, both in the general population and clinical settings (1). Hopelessness increases the risk of emotional maladjustment (2). The literature has consistently shown that hopelessness is a risk factor for depression (3). Individuals with depression experience higher levels of hopelessness than those without depression (4–6). Whether or not ethnic groups differ in the link between depression and hopelessness is, however, unknown.

Negative perspectives concerning the future, which is hopelessness, is a part of Beck’s negative cognitive triad (7). Hopelessness is a main aspect of a depressive cognitive style (7). As described by Beck, hopelessness plays a key role in the trajectory of depression and suicide, and it has been confirmed as a risk factor for negative outcomes associated with depression (8, 9). Both retrospective and large-scale prospective follow-up studies have documented hopelessness as a strong predictor of suicidal behavior (10, 11). Hopelessness also predicts general mood outcomes and functioning in the presence of affective disorders (12).

Ethnicity influences how hopelessness (13) and depression (14) are distributed across populations. Studies have shown that hope and optimism are differently experienced across ethnic groups, who are different in cultural experiences, life circumstances, values, and beliefs (15–17). Hirsch et al. studied depressive symptoms, hopelessness [defined as future attitudes and motivation loss (pathways)], trait hope [defined as confidence in the ability to identify and attain goals (agency)], and suicidal behaviors among Whites and Blacks. Trait hope buffered the association between low depressive symptoms and suicidal behavior for Whites but not Blacks. In addition, while hope remained a significant moderator for Whites, hopelessness operated as the main buffer for Blacks. The study suggested that outcomes associated with hope and hopelessness differ based on ethnicity (13).

*Differential effect hypothesis* views ethnicity not only as a proxy of exposure to risk and protective factors but also as a contextual factor that shapes human’s resilience and vulnerability. Based on this view, ethnicity operates as a contextual factor and alters how the very same risk and protective factors influence the very same health outcomes across diverse populations (18–25). This view is very different from the traditionally used *differential exposure hypothesis*, which attributes disparities in outcomes to group differences in exposures rather than vulnerabilities. Distinct from the *differential exposure hypothesis*, which assumes universality of the effects of risk and resilience factors, the *differential effect hypothesis* is focused on heterogeneity in the magnitude and direction of the effects that ultimately shape health and illness of diverse populations. Thus, instead of merely focusing on the main effect of ethnicity on exposures and outcomes or testing mediating effects of exposure, the *differential effect hypothesis searches for* qualitative and quantitative differences in the magnitude and direction of the associations between predictors and outcomes (26). According to the *differential effect hypothesis*, associations in health are seldom universal but specific to context (22–26).

Using a national sample of older adults in the United States and built on previous studies in the field (13)^1^^,^^2^ the current study tests the moderating effect of ethnicity on the association between depressive symptoms and hopelessness. Based on previous theoretical work (27–29) and empirical evidence (1–6) on hopelessness and depression, we expected a weaker association between depressive symptoms and hopelessness for Blacks compared to Whites, given their higher levels of religiosity (30–32), connectedness (33), and social support (34). This study contributes to the literature as – up to our knowledge – very limited information exists on group differences in the association between depression and hopelessness.

## Materials and Methods

### Design and Setting

This cross-sectional study used baseline data of the religion, aging, and health survey, a household survey, 2001. The study protocol received Institutional Review Board (IRB) approval from University of Michigan. All participants provided consent (35).

### Sampling and Participants

The study is limited to White or Black American older adults. All participants were non-institutionalized English speaking people of age more than 65 years. The study population was limited to Christians or those who were never associated with any faith. Older Blacks were oversampled in the survey (35).

### Measures

Ethnicity, age, gender, education, marital status, self-rated health (SRH), depressive symptoms, and hopelessness were measured *via* face-to-face interviews in 2001.

#### Depressive Symptoms

An eight-item Center for Epidemiological Studies-Depression scale (CES-D) (36) was used to measure depressive symptoms. Items used were as the following: (1) felt I could not shake off the blues even with the help of my family and friends, (2) I felt depressed, (3) I had crying spells, (4) I felt sad, (5) I did not feel like eating, my appetite was poor, (6) I felt that everything I did was an effort, (7) My sleep was restless, and (8) I could not get going. All these items either measure negative affect or somatic symptoms. Positive affect and interpersonal items were not reflected in this version of the CES-D. Abbreviated CES-D measures using 8 items (37, 38) have shown acceptable reliability and validity as compared to the original 20-item version (27, 39, 40). Responses ranged from 1 (“rarely or none”) to 4 (“most or all of the time”). We calculated mean score which was treated as a continuous measure, with a potential range from 1 to 4. Higher scores indicated more depressive symptoms (Alpha = 0.870 for all, 0.849 for Whites, 0.889 for Blacks).

#### Hopelessness

Hopelessness was measured with the following four items: (1) I always look on the bright side of things, (2) I am optimistic about my future, (3) In uncertain times, I always expect the best, and (4) I feel confident that the rest of my life will turn out well. These items are based on the scales developed by Scheier and Carver (3) and Krause (41). Item responses included strongly agree (1), agree (2), disagree (3), and strongly disagree (4). We did not use any specific cut-off score. Instead, we operationalized hopelessness as a continuous measure. We calculated our hopelessness score as the mean of the four items, where a higher score reflected more hopelessness (less hope) (42) (Alpha = 0.896 for all, 0.892 for Whites, 0.901 for Blacks).

#### Self-Rated Health

Individuals were evaluated for their SRH in 2001 using the following single item: how would you rate your overall health at the present time? Would you say your health is excellent, good, fair, or poor? Response options included excellent (1), good (2), fair (3), or poor (4). SRH has shown high reliability and validity. We operationalized SRH as a dichotomous variable comparing poor vs. other statuses. Dichotomization of a continuous (ordinal) variable will result in loss of information; however, SRH has been widely treated as a dichotomous variable in the literature (43–45). Operationalizing SRH as a dichotomous variable increases the degrees of freedom in our models.

### Statistical Analysis

We used SPSS 20.0 for Windows (IBM Inc., Armonk, NY, USA) for data analysis. For bivariate analysis, we used Pearson correlation test. For multivariable analysis, linear regression was applied. To test the reciprocal associations between depressive symptoms, once we considered depressive symptoms as an independent variable and hopelessness as the outcome, then we ran linear regressions with hopelessness as the independent variable and level of depressive symptoms as the outcome. In the first step, we fitted linear regressions in the pooled sample (*Model 1*). Then, we added the interaction term (*Model 2*), which was the multiplication of ethnicity (0 for Whites 1 for Blacks) and depressive symptoms or hopelessness (treated as a continuous measure). In the next step, we ran ethnic-specific models (*Model 4* and *Model 5*). In all our models, demographics, socioeconomics, and SRH were covariates, and ethnicity was the moderator. Adjusted correlation coefficients (β), SE, and 95% Confidence Intervals (CI) were reported. *p*-Values <0.05 were considered statistically significant.

## Results

Table 1 presents descriptive statistics, overall and also based on race. While age was not significantly different between Blacks and Whites (74.91 ± 6.49 vs. 75.37 ± 6.82, *p* > 0.05), Blacks were more likely to be female (65.1 vs. 58.6%, *p* < 0.05), had lower education (44.0 vs. 73.4%, *p* < 0.05), were less frequently married (35.7 vs. 59.5%, *p* < 0.05), and reported worse self-rated health (15.1 vs. 8.2%, *p* < 0.05) (for all comparisons). Blacks had marginally significant higher depressive symptoms (1.59 ± 0.65 vs. 1.54 ± 0.59, *p* = 0.06). Hopelessness was not different between Blacks and Whites (1.82 ± 0.60 vs. 1.91 ± 0.58, *p* > 0.05).

**Table 1:** **Descriptive statistics in the pooled sample and based on ethnicity**.

	All		Whites		Blacks	
	Mean	SD	Mean	SD	Mean	SD
Age	75.14	6.66	75.37	6.82	74.91	6.49
Depressive symptoms^#^	1.57	0.62	1.54	0.59	1.59	0.65
Hopelessness	1.87	0.59	1.91	0.58	1.82	0.60
Self-rated health (poor)*	1.55	0.64	1.53	0.65	1.58	0.63

	***n***	**%**	***n***	**%**	***n***	**%**

Gender*						
Male	570	38.2	314	41.4	256	34.9
Female	923	61.8	445	58.6	478	65.1
Education (high school diploma)*						
Yes	872	59.0	552	73.4	320	44.0
No	607	41.0	200	26.6	407	56.0
Marital status (married)*						
No	773	52.2	306	40.5	467	64.3
Yes	708	47.8	449	59.5	259	35.7
Self-rated health*						
Fair, good, or excellent	1316	88.4	694	91.8	622	84.7
Poor	173	11.6	62	8.2	111	15.1

*^#^*p* < 0.1*.

*p < 0.05.

Table 2 shows the results of correlations between study variables in the pooled sample as well as ethnic groups. Based on this table, depressive symptoms and hopelessness were positively correlated in the pooled sample (*r* = 0.151, *p* < 0.001). Depressive symptoms and hopelessness were correlated among Whites (*r* = 0.227, *p* < 0.001) but not Blacks (*r* = 0.080, *p* > 0.05).

**Table 2:** **Bivariate correlation matrix in the pooled sample and based on ethnicity**.

	1	2	3	4	5	6	7	8
**All**								
1 Ethnicity (Blacks)	1	−0.034	0.067**	−0.299**	−0.238**	0.108**	0.048	−0.006
2 Age		1	0.023	−0.109**	−0.177**	0.107**	0.027	0.061
3 Gender (women)			1	−0.039	−0.321**	0.019	0.084**	0.046
4 Education (high school diploma)				1	0.170**	−0.164**	−0.150**	−0.049
5 Marital status (married)					1	−0.078**	−0.120**	−0.047
6 Self-rated health (poor)						1	0.300**	0.142**
7 Depressive symptoms							1	0.151**
8 Hopelessness								1
**Whites**								
1 Ethnicity (Blacks)	–	–	–	–	–	–	–	–
2 Age		1	0.081*	−0.126**	−0.237**	0.136**	0.078*	0.087
3 Gender (women)			1	−0.067	−0.317**	0.036	0.101**	0.042
4 Education (high school diploma)				1	0.154**	−0.125**	−0.175**	−0.034
5 Marital status (married)					1	−0.067	−0.146**	−0.079
6 Self-rated health (poor)						1	0.267**	0.131**
7 Depressive symptoms							1	0.227**
8 Hopelessness								1
**Blacks**								
1 Ethnicity (Blacks)	–	–	–	–	–	–	–	–
2 Age		1	−0.029	−0.124**	−0.144**	0.086*	−0.013	0.040
3 Gender (women)			1	0.020	−0.315**	−0.002	0.059	0.052
4 Education (high school diploma)				1	0.067	−0.151**	−0.116**	−0.076
5 Marital status (married)					1	−0.042	−0.082*	−0.013
6 Self-rated health (poor)						1	0.324**	0.157**
7 Depressive symptoms							1	0.080
8 Hopelessness								1

*p < 0.05.

**p < 0.01.

### Effect of Depressive Symptoms on Hopelessness

Table 3 shows the results of four linear regressions with depressive symptoms as the independent and hopelessness as the dependent variable. *Model 1* and *Model 2* are run in the pooled sample, *Model 3* was run among Whites, and *Model* 4 among Blacks. *Model 1*, which did not include any interaction, showed a positive association between depressive symptoms and hopelessness (β = 0.12, 95% CI = 0.33–1.17), net of all covariates. *Model 2* showed a significant interaction between ethnicity and depressive symptoms on hopelessness (β = −0.25, 95% CI = −1.83 to 0.22, for the interaction term), suggesting a larger effect for Whites compared to Blacks. This interaction was significant net of all covariates. According to *Model 3*, depressive symptoms were positively associated with hopelessness among Whites (β = 0.20, 95% CI = 0.72–1.93). Depressive symptoms were not associated with hopelessness among Blacks (β = 0.04, 95% CI = −0.39 to 0.81) (*Model 4*).

**Table 3:** **Summary of the linear regressions for the association between depressive symptoms (independent variable) and hopelessness (dependent variable) in the pooled sample and based on race**.

	β (SE)	95%CI	β (SE)	95%CI	β (SE)	95%CI	β (SE)	95%CI
	
	All				Whites		Blacks	
	Model 1 Main effect		Model 2 With interaction		Model 3		Model 4	
Race (Blacks)	−0.04 (0.26)	−0.81–0.22	0.17 (0.68)^#^	−0.06–2.59	–	–	–	
Age	0.06 (0.02)^#^	0.00–0.08	0.06 (0.02)^#^	0.00–0.08	0.07 (0.03)	−0.01–0.10	0.05 (0.03)	−0.03–0.09
Gender (female)	0.04 (0.26)	−0.20–0.84	0.04 (0.26)	−0.19–0.85	0.02 (0.35)	−0.53–0.83	0.07 (0.41)	−0.25–1.35
Education (graduated from high school)	−0.02 (0.27)	−0.67–0.38	−0.02 (0.27)	−0.68–0.38	0.02 (0.40)	−0.63–0.96	−0.05 (0.36)	−1.10–0.33
Marital status (married)	−0.01 (0.27)	−0.58–0.48	0.00 (0.27)	−0.54–0.52	−0.03 (0.37)	−0.96–0.49	0.03 (0.40)	−0.56–1.02
Self-rated health (poor)	0.10 (0.46)**	0.43–2.25	0.11 (0.47)**	0.54–2.36	0.08 (0.71)^#^	−0.22–2.58	0.13 (0.62)**	0.40–2.85
Depressive symptoms	0.12 (0.22)***	0.33–1.17	0.21 (0.31)***	0.69–1.89	0.20 (0.31)***	0.72–1.93	0.04 (0.30)	−0.39–0.81
Blacks × depressive symptoms	–	–	−0.25 (0.41)*	−1.83–0.22				

*^#^*p* < 0.1*.

*p < 0.05.

**p < 0.01.

***p < 0.001.

### Effect of Hopelessness on Depressive Symptoms

Table 4 shows the results of four linear regression models with hopelessness and depressive symptoms as the independent and dependent variables, respectively. The first two linear regressions are in the pooled sample, the third model is among Whites, and the fourth model is among Blacks. *Model 1*, which did not include the interaction term, showed hopelessness is positively associated with depressive symptoms, above and beyond all covariates (β = 0.11, 95% CI = 0.01–0.03). *Model 2* showed a significant interaction between ethnicity and hopelessness on depressive symptoms (β = −0.27, 95% CI = −0.04 to 0.00), suggesting a stronger association for Whites compared to Blacks. This interaction was significant net of all covariates. *Model 3* showed that hopelessness was associated with depressive symptoms among Whites (β = 0.19, 95% CI = 0.02–0.04). Hopelessness was not associated with depressive symptoms among Blacks (β = −0.02, 95% CI = −0.01 to 0.03) (*Model 4*).

**Table 4:** **Summary of the linear regressions for the association between hopelessness (independent variable) and depressive symptoms (dependent variable) in the pooled sample and based on race**.

	β (SE)	95%CI	β (SE)	95%CI	β (SE)	95%CI	β (SE)	95%CI
	
	All				Whites		Blacks	
	Model 1 Main effect		Model 2 With interaction		Model 3		Model 4	
Race (Blacks)	−0.01 (0.04)	−0.09–0.06	0.24 (0.14)*	0.00–0.56				
Age	−0.06 (0.00)^#^	−0.01–0.00	−0.06 (0.00)^#^	−0.01–0.00	−0.05 (0.00)	−0.01–0.00	−0.05 (0.00)	−0.01–0.00
Gender (female)	0.04 (0.04)	−0.03–0.13	0.04 (0.04)	−0.03–0.13	0.03 (0.05)	−0.07–0.13	0.06 (0.07)	−0.05–0.21
Education (graduated from high school)	−0.11 (0.04)***	−0.22–0.05	−0.11 (0.04)***	−0.22–0.06	−0.15 (0.05)***	−0.28–0.07	−0.04 (0.06)	−0.18–0.07
Marital status (married)	−0.11 (0.04)**	−0.21–0.04	−0.10 (0.04)**	−0.20–0.04	−0.10 (0.06)*	−0.25–0.02	−0.11 (0.06)*	−0.25–0.02
Self-rated health (poor)	0.26 (0.07)***	0.43–0.70	0.26 (0.07)***	0.43–0.70	0.18 (0.10)***	0.22–0.63	0.32 (0.09)***	0.48–0.85
Hopelessness	0.11 (0.01)***	0.01–0.03	0.18 (0.01)***	0.01–0.04	0.19 (0.01)***	0.02–0.04	0.02 (0.01)	−0.01–0.03
Blacks × hopelessness			−0.27 (0.01)*	−0.04–0.00				

*^#^*p* < 0.1*.

*p < 0.05.

**p < 0.01.

***p < 0.001.

## Discussion

Our study documented stronger reciprocal associations between depressive symptoms and hopelessness among Whites compared to Blacks. Our findings are in line with the *differential effect hypothesis*, which suggests ethnic groups differ for the associations that ultimately influence health and illness (26, 46–49). That is, ethnicity shapes the degree by which depressive symptoms accompany hopelessness in the general population of older adults.

We found a positive association between depressive symptoms and hopelessness in the pooled sample and Whites. Hopelessness positively correlates with depression (50–52) and suicidality (53) and negatively correlates with happiness (2) and appropriate adjustment in the presence of stress (54, 55). Hopelessness is a core feature in depressed individuals (1–6). Compared with individuals who are hopeless, those who maintain hope are more resilient when faced with stress (39). Hope serves as a motivational factor and helps with initiation and sustaining actions toward goals (56). In the context of clinical depression, hopelessness is a main determinant of suicidality (57), compliance, and treatment outcomes (58).

Our findings on the moderating effect of ethnicity on the depression–hopelessness link suggest that Beck’s cognitive triad of depression may operate differently for sub-populations. Based on Beck’s Cognitive Theory of Depression, negative cognitions about the future (hopelessness), as well as dysfunctional beliefs about self and the world, are core components of depression (2). Our study suggests that hoplessness accompanies depression differently across ethnic groups. Black–White differences were observed in the effects of depression on dysfunctional attitudes about self^1,2^. In two nationally representative studies using independent samples, Assari has documented a stronger effects of depression on self-esteem and self-evaluation among Blacks compared to Whites^1^. These studies have suggested that depression may leave a larger scar on self-perception for Blacks compared to Whites^1^.

Our findings on ethnic-specific attitudes about future (hopelessness) may inform how cognitive behavioral therapy and psychotherapy can benefit from tailoring based on ethnicity. Dysfunctional attitudes about future, self, and others are being considered as targets of psychotherapy of the illness (59). Ethnic groups, who differ in how depression changes attitudes about self, others, and future (60), may benefit differently from cognitive behavioral therapies with the same level of emphasis on attitudes about self, others, and future. Our results suggest that Blacks may need less emphasis on hope as a component of psychotherapy for depression, compared to Whites (61). Thus, current results are relevant to the program planning and clinical practice that aim to reduce ethnic inequalities in depression outcomes in the United States. Findings also inform how we should measure depression (as a latent factor) is composed of dysfunctional attitudes about future, self, and others, across ethnically diverse groups (62). This is particularly important because such dysfunctional attitudes are prognostic factors for the treatment of depression (63).

Although early theories of hope had conceptualized the construct as a unidimensional motivational force (28), more recent models have expanded how we define hope. In 1969, Stotland described hope as “an expectation greater than zero of achieving a goal” (29). In 1987, Scheier and Carver defined hope and optimism as the “tendency to believe that one will generally experience positive vs. negative outcomes in life” (27, 64). Snyder viewed hope as an “internal sense of success and an active motivation state” (3, 65, 66). In Snyder’s view, hope is composed of two interrelated cognitive dimensions: agency and pathways (3, 65–67). Agency and pathway components of hope have different implications regarding depression (28). In one study, the agency but not the pathway component of hope predicted later depression (28).

Our findings also suggest that the salience of hopelessness as a mechanism behind development and course of depression may vary across ethnic groups. According to the theory of hopelessness depression, “hopelessness temporally precedes depression and is a proximal sufficient cause of the symptoms of depression.” Based on this theory, hopelessness explains the effects of stress and lack of social support on depression (68). Our findings suggest that the degree by which hopelessness contributes to depression is larger for Whites than Blacks (69). Whether or not the theory of hopelessness depression is differently valid for Blacks and Whites is still unknown.

Our findings on Black–White differences in the link between depressive symptoms and hopelessness are in line with other Black–White differences in other domains of health, which are linked with depression. Blacks and Whites differ in the links between stress, medical conditions, and depression (14, 49, 70– 72). Blacks and Whites also differ in the effect of baseline depressive symptoms on subsequent medical conditions and death (3, 14, 24, 73, 74). These differential associations can be explained by our current finding that ethnicity mitigates the link between depressive symptoms and hope. The results also support Black–White paradox (74, 75), defined as a lower risk of depression among Blacks despite a higher risk of adversities including medical conditions (14, 71, 76).

Our findings are not supported by the literature that suggests clinical depression is more consequential for Blacks compared to Whites (14, 77, 78). Depression is believed to be more chronic and disabling for Blacks compared to Whites (14, 77, 78). Higher prevalence of comorbid medical conditions (79, 80), somatic presentation of depression (81), lower access and trust to the health-care system combined with higher stigma (14, 77, 78), as well as negative beliefs regarding pharmaceutical treatments (14, 82) make depression more consequential for Blacks compared to Whites (14, 83, 84). Blacks have a lower chance of diagnosis and treatment of depression, and if they do receive a diagnosis, they will receive lower quality of services as they tend to get depression treatment in primary care settings rather than mental health-care settings (78, 85).

Our findings do not support the multiple disadvantage theory (86–88), which suggests the same risk factors are more harmful for Blacks than Whites, as they are exposed to multiple exposures (89). Our findings that depressive symptoms accompanied more hope for Blacks compared to Whites can be better understood in a resiliency framework (90). Despite a higher rate of poverty (91), lower social position (78), more severe depression (92), more comorbid conditions (78), less access to health care (72), and differences in the diagnosis and treatment of depression (93), Blacks with depression may have a higher tendency to maintain hope, possibly due to endorsing higher levels of religiosity and receiving higher levels of social support.

Our findings have major implications for clinical practice. Sense of hope is essential for maintaining self-efficacy (5), self-care, health services use, and positive psychological outcomes (94). Compared to Whites, Blacks with depression see the future as more positive, a finding that may help clinicians who treat depression among diverse population groups. Hopelessness increases negative feelings and uncertain views about future events and possibilities (67) and reduces the expectation that positive outcomes may be forthcoming in the future (3, 66, 95–97). These negative views may be more prevalent in depression among Whites than Blacks.

An implication of our finding is for suicide research and practice. Hopelessness strongly predicts suicide and may mediate the effect of depression on suicidality (98). Thus, our findings on higher hopefulness in the presence of depression may partially explain the lower suicide rate of Blacks compared to Whites (46, 99, 100). According to our findings, suicide prevention programs among Whites who suffer depression may require more emphasis on hope enhancement (101).

Our study had a few limitations. The first limitation is the exploratory nature of our statistical analyses conducted. Second, validity of measures used to assess depressive symptoms and hopelessness may vary across ethnic groups (102). We did not use specific psychometric tools to assess hopelessness but simply adopted a four-item scale. We cannot rule out differential underreporting of depressive symptoms and hopelessness across ethnic groups (81, 103). We also did not measure stereotype threat. Blacks may express less hopelessness due to concerns regarding being negatively evaluated. Third, participants were either Christians or those who were never associated with any faith. Future research should test if ethnicity or faith explains Black–White differences in the link between depression and hopelessness. Finally, as we did not have longitudinal data and did not use path modeling for data analysis, we do not conclude any causation from observed associations. Thus, we do not argue that depression differently causes hopelessness across groups. Both empirical evidence and theoretical work have suggested that the links between depression and hopelessness are bidirectional. Based on the *scar model*, depression diminishes hope and according to the *vulnerability model*, hopelessness increases vulnerability for depression (4–6). Despite these limitations, our study is one of the first studies on ethnic heterogeneity of the association between depression and hopelessness. Using a national sample and considerable sample size of Blacks were the strengths of the current study.

Results of this study help us understand ethnic differences in correlates of depression. Hope is one of the most neglected virtues in the literature (104). Human virtues such as hope and optimism are strong buffers against psychological adversities on a wide range of mental disorders (105, 106).

More research is needed on possible roles that attitudes about self, others, and future play on depression across various ethnic groups (103). Future research may also explore how the current findings can be used to reduce the burden of depression and hopelessness across populations. Differential hopelessness may contribute to ethnic variation in causes, courses, and consequences of depression across ethnic groups, which have implications for the elimination of health disparities (107–111).

## Conclusion

In summary, Black–White differences exist in the magnitude of the association between depressive symptoms and hopelessness, with Blacks showing a weaker association compared to Whites. This finding suggests that depression may be more hopeless for Whites compared to Blacks.

## Ethics Statement

All procedures followed were in accordance with the ethical standards of the responsible committee on human experimentation (institutional and national) with the Helsinki Declaration of 1975, as revised in 2000. Informed consent was obtained from all participants included in the study.

## Informed Consent

Informed consent was obtained from all participants included in the study.

## Author Contributions

SA designed and analyzed this work, and ML drafted and revised the paper. SA had full access to all of the data in the study and takes responsibility for the integrity of the data and the accuracy of the data analysis.

## Conflict of Interest Statement

The authors declare that the research was conducted in the absence of any commercial or financial relationships that could be construed as a potential conflict of interest.

## Footnotes

^1^Assari S, Dejman M. Heterogeneity of the depression – self-esteem association based on race and gender. *Front Psychol* (2016).

^2^Assari S. Link between depressive symptoms and self-evaluation stronger among Blacks than Whites. *Front Hum Neurosci* (2016).
